# Capped Linex Metric Twin Support Vector Machine for Robust Classification

**DOI:** 10.3390/s22176583

**Published:** 2022-08-31

**Authors:** Yifan Wang, Guolin Yu, Jun Ma

**Affiliations:** School of Mathematics and Information Science, North Minzu University, Yinchuan 750021, China

**Keywords:** capped linex loss function, robustness, classification, outliers

## Abstract

In this paper, a novel robust loss function is designed, namely, capped linear loss function $L_{a\varepsilon }$. Simultaneously, we give some ideal and important properties of $L_{a\varepsilon }$, such as boundedness, nonconvexity and robustness. Furthermore, a new binary classification learning method is proposed via introducing $L_{a\varepsilon }$, which is called the robust twin support vector machine (Linex-TSVM). Linex-TSVM can not only reduce the influence of outliers on Linex-SVM, but also improve the classification performance and robustness of Linex-SVM. Moreover, the effect of outliers on the model can be greatly reduced by introducing two regularization terms to realize the structural risk minimization principle. Finally, a simple and efficient iterative algorithm is designed to solve the non-convex optimization problem Linex-TSVM, and the time complexity of the algorithm is analyzed, which proves that the model satisfies the Bayes rule. Experimental results on multiple datasets demonstrate that the proposed Linex-TSVM can compete with the existing methods in terms of robustness and feasibility.

## 1. Introduction

Data collecting and reasonable processing are becoming increasingly crucial as modern computer technology advances. As an excellent machine learning tool, support vector machine (SVM) [1,2,3,4] has been widely used in financial forecast, bioinformatics, computer vision, image annotation, data mining and other fields in recent years. The main idea of SVM classification based on statistical learning theory and optimization theory is to construct a pair of parallel hyperplanes to maximize the minimum distance between two classes of samples. Generally speaking, the optimal hyperplane is realized by solving an optimization problem with inequality constraints. In order to avoid overfitting, scholars extend support vector machines to soft difference support vector machines (C-SVM) [5], introduce relaxation variables to relax constraints, and increase the penalty term of relaxation variables in the objective function. However, the loss function adopted by C-SVM is generally a hinge loss, which makes it very sensitive to noise. In the following research, C-SVM is extended to deal with the problem of function estimation, and a support vector interpretation of ridge regression [6] is proposed, which is different from the inequality constraints in C-SVM, as it uses equivalent constraints. Similarly, Suykens [7] considered equality constraints in the sense of least squares and proposed a least squares support vector machine (LSSVM). Unlike C-SVM, it does not use the non-support vector machine to optimize the classifier; LSSVM makes full use of the information of all data points and uses $L_{2}$ loss to punish both data points symmetrically. In order to further improve the performance of classification, researchers need to impose heavier penalties on samples that are misclassified. For this reason, in the literature [8], Ma et al. considered asymmetric linear exponential loss (LINEX) to be used to achieve this goal, and Linex-TSVM is proposed to study the binary classification problem. However, both SVM, C-SVM, LSSVM and Linex-TSVM have their own advantages, but they all have to solve a large-scale QPP(Quadratic Programming problem), which requires a lot of time to learn and is not suitable for dealing with practical problems.

Because all the above models need to solve a big QPP, to further improve the computing speed, Jayadeva et al. [9] proposed a twin support vector machine (TSVM) for pattern classification based on the generalized eigenvalue approximation support vector machine (GEPSVM). Since TSVM solves two smaller QP problems instead of a single large QPP, it can theoretically learn four times faster than a standard SVM. The main goal of TSVM is to find two parallel hyperplanes, each of which is as close as possible to the corresponding class in the sample data, while being as far away from the other classes as possible. Therefore, TSVM is more suitable for the classification of large-scale data.

It is well known that distance metrics play a crucial role in many machine learning algorithms. Although the above algorithms demonstrate good performance in pattern classification, it is worth noting that most of them adopt the $L_{2}$-norm distance metric, whose squaring operation will exaggerate the impact of outliers on model performance. To effectively alleviate the impact of the $L_{2}$-norm distance metric on the robustness of the algorithm, the $L_{1}$-norm distance metric with the bounded derivative has received extensive attention and research in many fields of machine learning in recent years [10,11,12]. Recently, more and more researchers have paid attention to the capped $L_{1}$-norm. The capped $L_{1}$-norm can solve the deficiency of $L_{1}$-norm unboundedness. In particular, Wang et al. [13] proposed a new robust TSVM (CTSVM) by the applying the capped $L_{1}$-norm.

Inspired by the successful application of capped $L_{1}$-norm and linex loss function [14,15,16,17], meanwhile, the latest research shows that no scholar has extended the Linex loss function to twin support vector machines, therefore, a new robust twin support vector machine is established in this paper. The details and the main contributions of this work are as follows:

(1) A novel robust loss function is designed, namely, capped linear loss function $L_{a\varepsilon }$.

(2) A novel robust twin support vector machine, namely capped linex twin support vector machine (Linex-TSVM) is proposed.

(3) A efficient iterative algorithm is designed to solve Linex-TSVM, which is not only easy to implement, but also theoretically guarantees the existence of a reasonable optimal solution. We analyze the computational complexity of the algorithm and prove that the model satisfies the Bayesian rule.

(4) Extensive experiments conducted across multiple datasets demonstrates that the proposed Linex-TSVM is competitive with state-of-the-art methods in terms of robustness and feasibility. Therefore, the Linex-TSVM is feasible for practical applications.

The rest of this article is organized as follows. In Section 2, we briefly review Linex-SVM and TSVM. In Section 3, we describe in detail the proposed capped linex loss function and Linex-TSVM, and give the relevant theoretical analysis. After the experimental results on multiple data sets are presented in Section 4, we conclude this paper in Section 5.

## 2. Related Work

In this section, we are warranted to review Linex-SVM and TSVM.

### 2.1. Linex-SVM

Linex loss function is a typical asymmetric loss function, defined as:

(1) $$\begin{array}{c} L_{linex}\left(x\right)=e^{ax}-ax-1 \end{array}$$

where a≠0 is a parameter. If a<0, the left side of Linex loss is steeper than the right side, and the opposite is true when a>0. The value of |a| decides the symmetry of Linex loss function. This shows that the symbol of a determines the shape of the function. When *a* takes an appropriate value, it can be reduced to square loss. Linex loss function is not only asymmetric, but also convex and derivable; thus, it is widely used in statistics.

For the dichotomy problem in n-dimensional Euclidean space, the training set can be expressed as

(2) $$\begin{array}{c} T=\{\left(x_{1},y_{1}\right),\left(x_{2},y_{2}\right),\cdots ,\left(x_{m},y_{m}\right)\} \end{array}$$

where $x_{i}\in R^{n}$ is the feature vector of the data *i*, and $y_{i}\in \left\{-1,+1\right\}$ is the label of the data *i*.

For the training set Equation (2), the Linex-support vector machine model can be written as a convex optimization problem with equation constraints in Equation (3) by introducing a linear loss function:

(3) $$\begin{array}{cc} \; & \min_{\omega ,b,\xi }\;\;\frac{1}{2}{∥\omega ∥}^{2}+C\sum_{i=1}^{m}\left(e^{a\xi_{i}}-a\xi_{i}-1\right),\\ \; & s.t\;\;\;y_{i}\left(\omega^{T}x_{i}+b\right)-1=\xi_{i},\;i=1,2,\cdots ,m. \end{array}$$

where $\xi =(\xi_{1},\xi_{2},\cdots ,\xi_{m})$ is a slack variable, *C* is a penalty parameter and *a* is a parameter of the Linex loss. Furthermore, we can use the Nesterov accelerated gradient (NAG) method to obtain the optimal solution $\left(\omega_{1},b_{1}\right)$ and construct the decision function $f\left(x\right)=sgn\left(\omega_{1}^{T}x+b_{1}\right)$.

### 2.2. TSVM

The support vector machine is not suitable for dealing with large-scale data, to improve the practical application of the model and further shorten the learning time. Jayadeva et al. proposed a twin support vector machine (TSVM) for pattern classification based on the generalized eigenvalue approximation support vector machine (GEPSVM). The details are as follows:

Considering *n* dimensional Euclidean space $R^{n}$ the binary classification problem, the training set is $T=\{x_{i},y_{i}|i=1,2...m\}$, $x_{i}\in R_{n}$, where $y_{i}\in \left\{-1,1\right\}$. $A\in R^{m_{1}\times n}$ represents all positive samples; $B\in R^{m_{2}\times n}$ represents all negative samples. TSVM identifies two non-parallel hyper planes in the feature space:

(4) $$\begin{array}{c} f_{1}\left(x\right)=\omega_{1}^{T}x+b_{1}=0, \end{array}$$

(5) $$\begin{array}{c} f_{2}\left(x\right)=\omega_{2}^{T}x+b_{2}=0. \end{array}$$

where $\omega_{1},\omega_{2}\in R^{n},b_{1},b_{2}\in R$.

The TSVM classifier is obtained by solving the following pair of QPPS:

(6) $$\begin{array}{cc} \; & \min_{\omega_{1},b_{1}}\;\;\frac{1}{2}{∥A\omega_{1}+e_{1}b_{1}∥}_{2}^{2}+C_{1}e_{2}^{T}\xi_{1},\\ \; & s.t\;\;\;-\left(B\omega_{1}+e_{2}b_{1}\right)+\xi_{1}\geq e_{2},\;\;\;\xi_{1}\geq 0. \end{array}$$

(7) $$\begin{array}{cc} \; & \min_{\omega_{2},b_{2}}\;\;\frac{1}{2}{∥B\omega_{2}+e_{2}b_{2}∥}_{2}^{2}+C_{2}e_{1}^{T}\xi_{2},\\ \; & s.t\;\;\;\left(A\omega_{2}+e_{1}b_{2}\right)+\xi_{2}\geq e_{1},\;\;\;\xi_{1}\geq 0. \end{array}$$

where $C_{1}\geq 0,C_{2}\geq 0$ represent regularization parameters, $e_{1},e_{2}$ are all unit vectors, $\xi_{1},\xi_{2}$ are the slack vectors.

Then, the dual problem of TSVM is obtained by dual theory:

(8) $$\begin{array}{cc} \; & \min_{\alpha }\;\;-\frac{1}{2}\alpha^{T}G{\left(H^{T}H+\lambda I\right)}^{-1}G^{T}\alpha +e_{2}^{T}\alpha ,\\ \; & s.t\;\;\;0\leq \alpha \leq C_{1}e_{2}. \end{array}$$

(9) $$\begin{array}{cc} \; & \min_{\beta }\;\;-\frac{1}{2}\beta^{T}H{\left(G^{T}G+\lambda I\right)}^{-1}H^{T}\beta +e_{2}^{T}\beta ,\\ \; & s.t\;\;\;0\leq \beta \leq C_{2}e_{1}. \end{array}$$

$\alpha \in R^{m_{2}}$ and $\beta \in R^{m_{1}}$ are lagrange multipliers. At the same time, matrix *G* and *H* respectively defined as: $G=[B\;e_{2}]$ and $H=[A\;e_{1}]$.

Furthermore, by introducing the kernel method, TSVM can be extended to nonlinear space, and it can be decided whether the sample data *x* belongs to positive class or negative class according to the shortest distance between the sample data *x* and two non-parallel planes. The decision function is

(10) $$f\left(x\right)=argmin_{k=1,2}\frac{|x\omega_{k}+b_{k}|}{∥\omega_{k}∥}.$$

## 3. Main Contribution

### 3.1. Capped Linex Loss Function

In this section, in order to minimize the influence of abnormal values on the classification results of the model, we propose a novel robust loss function, that is, the capped linex loss function. The details are as follows:

**Definition** **1.** *The capped linex loss function is defined as*

(11) $$L_{a\varepsilon }\left(x\right)=min\left(\sum_{i}e^{ax_{i}}-ax_{i}-1,\varepsilon \right)$$

**Figure 1:**
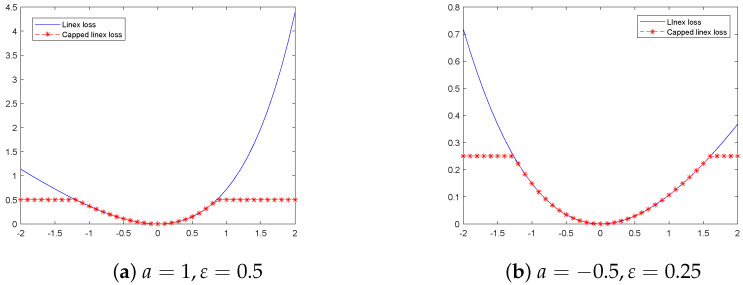
Linex loss capped linex loss.

*where a≠0 is a parameter, when a<0, the left side of the loss function is steeper than the right side; when a>0, the right side of the loss function is steeper than the left side; see Figure 1. ε>0 is a thresholding parameter; $x_{i}$ is the component of x.*

Figure 1 shows the comparison between the capped linex loss function and the linex loss function. Obviously, we can observe that the improved linear loss has an upper bound, and when the error tends to be consistent, even if there are outliers, the loss will not increase to a certain extent, which improves the robustness of the model.

### 3.2. Capped Linex Twin Support Vector Machine

Linex-SVM model still needs to be improved: linex loss is an unbounded function, and the loss tends to be consistent with the increase in error. However, in practical applications, datasets are often accompanied by noise, and the unboundedness of the linex loss function will affect the overall performance of the model. In other words, Linex-SVM is a relatively weak method in dealing with training sets with outliers. In addition, almost all the instances in the Linex-SVM contribute to the final optimal hyperplane, which will greatly reduce the training speed.

In order to improve the classification performance of Linex-SVM, we first improve Linex loss to capped linex loss and introduce regularization term to enhance robustness. Secondly, we generalize Linex-SVM to twin support vector machine, and transform a large QPP into two small QP problems to improve the training speed. Based on the above two points, a new twin support vector machine model, named the capped linex twin support vector machine (Linex-TSVM) is obtained:

(12) $$\begin{array}{cc} \; & \min_{\omega_{1},b_{1}}\sum_{i=1}^{m_{1}}\min(∥\omega_{1}x_{i}+b_{1}{∥}_{1},\varepsilon_{1})+C_{1}\sum_{i=1}^{m_{2}}\min\left(e^{a\xi_{i}}-a\xi_{i}-1,\varepsilon_{2}\right)+\frac{C_{3}}{2}(∥\omega_{1}{∥}_{2}^{2}+b_{1}^{2}),\\ \; & s.t\;\;-\left(B\omega_{1}+e_{2}b_{1}\right)+\xi \geq e_{2}. \end{array}$$

(13) $$\begin{array}{cc} \; & \min_{\omega_{2},b_{2}}\sum_{i=1}^{m_{2}}\min(∥\omega_{2}x_{i}+b_{2}{∥}_{1},\varepsilon_{3})+C_{2}\sum_{i=1}^{m_{1}}\min\left(e^{a\eta_{i}}-a\eta_{i}-1,\varepsilon_{4}\right)+\frac{C_{4}}{2}(∥\omega_{2}{∥}_{2}^{2}+b_{2}^{2}),\\ \; & s.t\;\;\left(A\omega_{2}+e_{1}b_{2}\right)+\eta \geq e_{1}. \end{array}$$

where $C_{1},C_{2},C_{3},C_{4}\geq 0$, $e_{1}\in R^{m_{1}}$ and $e_{2}\in R^{m_{2}}$ are the unit vectors. ξ and η are slack vectors.

In addition, we also notice that when using the traditional convex optimization method, it is difficult to solve the problems Equations (12) and (13) simply and quickly. Here, in order to simplify the original problem to an approximate problem that is easier to solve, we use the re-weighted trick [12,18,19,20], the most important of which is the formula ${∥x∥}_{1}=\frac{x^{T}x}{\left|x\right|}$. Take Equation (12) as an example, for the distance measurement items, when the $F=\frac{1}{\left|x\right|}$ holds, then ${∥x∥}_{1}=x^{T}Fx$. For the loss function terms, when $e^{a\xi_{i}}-a\xi_{i}-1\leq \varepsilon_{2}$, there are $\sum_{i=1}^{m_{2}}\min\left(e^{a\xi_{i}}-a\xi_{i}-1,\varepsilon_{2}\right)=\sum_{i=1}^{m_{2}}\left(e^{a\xi_{i}}-a\xi_{i}-1\right)$. Further, in order to simplify function $e^{a\xi }-a\xi -1$ into an easy-to-solve $\xi^{T}Q\xi$, we define *Q* as diagonal matrices with *i*-th diagonal element as:

(14) $$q_{i}=\left\{\begin{array}{c} \frac{e^{a\xi_{i}}-a\xi_{i}-1}{\xi_{i}^{2}},\;\;\;\;\;\;\;e^{a\xi_{i}}-a\xi_{i}-1\leq \varepsilon_{2}\\ 0,\;\;\;\;\;\;\;\;\;\;\;\;\;\;\;\;\;\;otherwise. \end{array}\right.$$

similarly,

(15) $$u_{i}=\left\{\begin{array}{c} \frac{e^{a\eta_{i}}-a\eta_{i}-1}{\eta_{i}^{2}},\;\;\;\;\;\;\;e^{a\eta_{i}}-a\eta_{i}-1\leq \varepsilon_{4}\\ 0,\;\;\;\;\;\;\;\;\;\;\;\;\;\;\;\;\;\;otherwise. \end{array}\right.$$

Based on the above discussion and calculation, we can obtain the optimization problem Equations (16) and (17), as follows:

(16) $$\begin{array}{cc} \; & \min_{\omega_{1},b_{1}}{\left(A\omega_{1}+e_{1}b_{1}\right)}^{T}F\left(A\omega_{1}+e_{1}b_{1}\right)+\frac{1}{2}C_{1}\xi^{T}Q\xi +\frac{C_{3}}{2}(∥\omega_{1}{∥}_{2}^{2}+b_{1}^{2}),\\ \; & s.t.\;\;-\left(B\omega_{1}+e_{2}b_{1}\right)+\xi \geq e_{2}. \end{array}$$

(17) $$\begin{array}{cc} \; & \min_{\omega_{2},b_{2}}{\left(B\omega_{2}+e_{2}b_{2}\right)}^{T}K\left(B\omega_{2}+e_{2}b_{2}\right)+\frac{1}{2}C_{2}\eta^{T}U\eta +\frac{C_{4}}{2}(∥\omega_{2}{∥}_{2}^{2}+b_{2}^{2}),\\ \; & s.t.\;\;\left(A\omega_{2}+e_{1}b_{2}\right)+\eta \geq e_{1}. \end{array}$$

where $e_{1}\in R^{m_{1}}$ and $e_{2}\in R^{m_{2}}$ are the unit vectors, *F* and *K* are also two diagonal matrices:

(18) $$f_{i}=\left\{\begin{array}{c} \frac{1}{|\omega_{1}x_{i}+b_{1}|},\;\;\;\;\;\;\;\left|\omega_{1}x_{i}+b_{1}\right|\leq \varepsilon_{1}\\ 0,\;\;\;\;\;\;\;\;\;\;\;\;\;\;\;\;\;\;otherwise. \end{array}\right.$$

(19) $$k_{i}=\left\{\begin{array}{c} \frac{1}{|\omega_{2}x_{i}+b_{2}|},\;\;\;\;\;\;\;\left|\omega_{2}x_{i}+b_{2}\right|\leq \varepsilon_{3}\\ 0,\;\;\;\;\;\;\;\;\;\;\;\;\;\;\;\;\;\;otherwise. \end{array}\right.$$

**Remark** **1.** *What is more detailed is that in the objective functions Equations* (16) *and* (17)*, we use diagonal matrices F, Q and K, U, respectively, to reduce the influence of outliers and abnormal noise on the model. Specifically, if the points in the same class are far away from the hyperplane, they can be treated as noise and removed. In addition, the model mainly sets the elements in the diagonal matrix according to the distance from the data point $x_{i}$ to the hyperplane. For F, if $f_{i}$ is greater than $\varepsilon_{1}$, the corresponding $f_{i}$ is set to a smaller value (Smallval), which is almost equivalent to 0. Where ’Smallval’ is a small constant, which will be set to 10^−8^ in the experiment.*

The corresponding Lagrange function of the above optimization problem Equation (16) can be written as:

(20) $$\begin{array}{ccc} L(\omega_{1},b_{1},\xi_{1},\alpha ) & = & \frac{1}{2}{\left(A\omega_{1}+e_{1}b_{1}\right)}^{T}F\left(A\omega_{1}+e_{1}b_{1}\right)+\frac{1}{2}C_{1}\xi^{T}Q\xi +\frac{C_{3}}{2}(∥\omega_{1}{∥}_{2}^{2}+b_{1}^{2})\\ & - & \alpha^{T}\left(-\left(B\omega_{1}+e_{2}b_{1}\right)+\xi -e_{2}\right) \end{array}$$

where α is a Lagrange multiplier, derive the Lagrange function about $\omega_{1}$ and $\beta_{1}$, obtain the following Karush–Kuhn–Tucker Conditions.

(21) $$\left\{\begin{array}{ccc} \frac{\partial L}{\partial \omega_{1}}=A^{T}F\left(A\omega_{1}+e_{1}b_{1}\right)+B^{T}\alpha +C_{3}\omega_{1}=0,\;\;\;\; & \left(i\right) & \\ \frac{\partial L}{\partial b_{1}}=e_{1}^{T}F\left(A\omega_{1}+e_{1}b_{1}\right)+e_{2}^{T}\alpha +C_{3}b_{1}=0,\;\;\;\; & \left(ii\right) & \\ \frac{\partial L}{\partial \xi_{i}}=C_{1}Q\xi \alpha =0,\;\;\;\;\;\; & \left(iii\right) & \\ \alpha^{T}\left(B\omega_{1}+e_{2}b_{1}-\xi +e_{2}\right)=0,\;\;\;\; & \left(iv\right) & \\ \alpha \geq 0.\;\;\;\; & \left(v\right) \end{array}\right.$$

Through KKT condition, the dual problem of Equation (12) is as follows:

(22) $$\begin{array}{cc} \; & \min_{\alpha }\frac{1}{2}\alpha^{T}\left(E{\left(H^{T}FH+C_{3}I\right)}^{-1}E^{T}+\frac{1}{C_{1}}Q^{-1}\right)\alpha -e_{2}^{T}\alpha ,\\ \; & s.t\;\;0\leq \alpha \leq C_{1}e_{2}. \end{array}$$

Similarly, the dual problem of Equation (13) is:

(23) $$\begin{array}{cc} \; & \min_{\alpha }\frac{1}{2}\beta^{T}\left(H{\left(E^{T}KE+C_{4}I\right)}^{-1}H^{T}+\frac{1}{C_{2}}U^{-1}\right)\beta -e_{1}^{T}\beta ,\\ \; & s.t\;\;0\leq \beta \leq C_{2}e_{1}. \end{array}$$

where β is a Lagrange multiplier, and

(24) $$H=\left[\begin{array}{cc} A & e_{1} \end{array}\right],\;E=\left[\begin{array}{cc} B & e_{2} \end{array}\right].$$

Thus, we get the vector $Z_{1}$ and $Z_{2}$, and gain the new data point $x\in R^{n}$ to a positive or negative category.

Based on the above discussion, our algorithm will be presented in Algorithm 1.
**Algorithm 1** Iterative algorithm to solve Linex-CTSVM**Input:** Training data $A\in R^{m_{1}\times n}$ and $B\in R^{m_{1}\times n}$; Parameters $C_{i}\left(i=1,2,3,4\right)$ and $\varepsilon_{i}\left(i=1,2,3,4\right)$. Establish matrixs $H=\left[A\;e_{1}\right],\;E=\left[B\;e_{2}\right]$. Initialize $F\in R^{m_{1}\times m_{1}}$ and $K\in R^{m_{2}\times m_{2}}$. Let k=0 Iterative
$${\left({\left[\omega_{1},b_{1}\right]}^{T}\right)}^{k+1}=-{\left(H^{T}FH+C_{3}I\right)}^{-1}E^{T}\alpha .$$
$${\left({\left[\omega_{2},b_{2}\right]}^{T}\right)}^{k+1}={\left(E^{T}KE+C_{4}I\right)}^{-1}H^{T}\beta .$$ Update matrix separately Q,U,F,K by Equations (14), (15), (18) and (19) Let *k* = *k* + 1 and go to step 2, until convergence stops. **Output:** Optimal solution ${\left[\omega_{1},b_{1}\right]}^{T}$ and ${\left[\omega_{2},b_{2}\right]}^{T}$.


### 3.3. Bayes Rule

We want to prove that the model proposed in this paper can satisfy the Bayes rule, assuming that the sample $\left(x_{i},y_{i}\right)$ are independent of the same probability ϕ, and the probability ϕ is defined on X×Y, where $X\in R^{n}$, Y=−1,1. Further, we assume that the conditional distribution ϕ(y|x) is a binomial distribution, including ϕ(−1|x) and ϕ(1|x). As we all know, the ultimate goal of the classification problem is to obtain a classifier C:X→Y with small error. Bayesian classifier [8] is defined as the classifier with the lowest probability of classification error among all kinds of classifiers.

(25) $$f_{C}\left(x\right)=\left\{\begin{array}{c} 1,\;\;\;\;if\phi (y=1|x)\geq \phi (y=-1|x),\\ -1,\;\;\;\;if\phi (y=1|x)<\phi (y=-1|x). \end{array}\right.$$

For any loss function *L*, the expected risk of the classifier f:X→R can be defined as

(26) $$\begin{array}{c} R_{L,\phi }=\int_{X\times Y}L\left(1-yf\left(x\right)\right)d\phi . \end{array}$$

Next, by minimizing the expected risk of all measurable classification functions, we can obtain

(27) $$\begin{array}{c} f_{L,\phi }=argmin_{\tau \in R}\int L\left(1-y\left(x\right)\tau \right)d\phi \left(y|x\right),\;\;\;\;\;\forall x\in X. \end{array}$$

Based on the above important definition of Bayes rule, we obtain Theorem 1 to prove that Bayes rule holds for capped linex loss function. The details of the proof are as follows.

**Theorem** **1.** *Function $f_{L_{a\varepsilon ,\phi }}$, which minimizes the expected risk on all measurable functions f:X→Y, making the result equivalent to that of a Bayes classifier, that is $f_{L_{a\varepsilon ,\phi }}\left(x\right)=f_{C}\left(x\right),\forall x\in X.$*

**Proof.**  By the properties of capped linex loss function, when $e^{ax_{i}}-ax_{i}-1<\varepsilon$, we can obtain

(28) $$\begin{array}{c} L_{a\varepsilon }\left(x\right)=e^{ax}-ax-1. \end{array}$$

So, there are

(29) $$\begin{array}{ccc} \int_{Y}L_{a\varepsilon }\left(1-y\left(x\right)\tau \right)d\phi \left(y|x\right) & = & L_{a\varepsilon }\left(1-\tau \right)\phi \left(y=1|x\right)+L_{a\varepsilon }\left(1+\tau \right)\phi \left(y=-1|x\right)\\ & = & e^{a(1-\tau )}-a\left(1-\tau \right)-1\phi \left(y=1|x\right)+e^{a(1+\tau )}-a\left(1+\tau \right)-1\phi \left(y=-1|x\right) \end{array}$$

By Equation (29), when ϕ(y=1|x)≥ϕ(y=−1|x) and ϕ(y=1|x)<ϕ(y=−1|x), obtain the minimum value at τ=−1 and τ=1, respectively, and when ϕ(y=1|x)=ϕ(y=−1|x), we obtain the minimum value at τ=−1 or τ=1. Therefore, when $e^{ax_{i}}-ax_{i}-1<\varepsilon$, the capped linex loss function can measure the minimum expected risk of $f_{L_{a\varepsilon ,\phi }}\left(x\right)$. To sum up

(30) $$f_{L_{a\varepsilon ,\phi }}\left(x\right)=\left\{\begin{array}{c} 1,\;\;\;\;if\phi (y=1|x)\geq \phi (y=-1|x),\\ -1,\;\;\;\;if\phi (y=1|x)<\phi (y=-1|x). \end{array}\right.$$

$\;i.e,\;\;f_{L_{a\varepsilon ,\phi }}\left(x\right)=f_{C}\left(x\right)$

□

### 3.4. Computational Complexity Analysis

This part mainly analyzes the computational complexity of Algorithm 1. As we all know, the computational complexity includes the number of iterations and the computational cost of iterations. The computational complexity of Algorithm 1 after one iteration is divided into two parts: (1) the time complexity of solving QPP is not more than $\frac{m^{3}}{4}$, and the inverse of matrix is not greater than (n+1)(n+1). Therefore, the total time complexity of solving Linex-TSVM is about $O(t\cdot \left(\frac{m^{3}}{4}\left(n+1\right)3\right))$, where *t* is the number of iterations, and the experimental results of this paper demonstrate that t=50 meets the expectation. Under the condition of universality, the number of iterations of each algorithm is much less than the number of samples. Similarly, Linex-TSVM has cubic time complexity in the number of samples.

## 4. Experimental Results and Discussions

In this section, we first set the experimental parameters in Section 4.1, and in sections Section 4.2 and Section 4.3, we give in detail the experimental results of the model Linex-TSVM with or without noise. Finally, we present some results on the data set in Section 4.4 to prove the convergence of the objective function.

### 4.1. Experimental Setup

#### 4.1.1. Evaluation Criteria

In order to evaluate the classification performance of our proposed truncated linear loss support vector machine more accurately, we compare it with other mature methods, including SVM, LSSVM, C-SVM, Linex-TSVM, and TBSVM. For these five support vector machines and Linex-TSVM, the iterative process is stopped when the difference between the target values of the two iterations is less than 0.001 and the number of iterations is more than 50. At the same time, in order to measure the performance of all algorithms, the traditional precision index (ACC) is used to measure the performance of these algorithms, which is defined as follows:

(31) $$\begin{array}{c} \mathrm{ACC}=\frac{\mathrm{TP}+\mathrm{TN}}{\mathrm{TP}+\mathrm{FN}+\mathrm{TN}+\mathrm{FP}}, \end{array}$$

Among them, TP and TN represent correct positive samples and negative samples, respectively. FN and FP represent wrong positive samples and negative samples, respectively. In order to make a more accurate comparison, we use the quadratic programming (QP) toolbox of matlab to solve the QP problem in related algorithms. The experimental environment consists of a Windows 10 machine and Intel i7 Processor (3.70 GHz) with 8 GB of RAM.

#### 4.1.2. Parameters Selection

For the learning algorithm, its performance is very sensitive to the parameters involved, so it is necessary to record the parameters of each algorithm in detail and list them as follows.

SVM and LSSVM:the kernel parameter σ.
C-SVM: the regularization parameter *c*, the kernel parameter σ.
NPSVM and TBSVM: the regularization parameters $c_{1}$, $c_{2}$, $c_{3}$ and $c_{4}$, the kernel parameter σ.
Linex-SVM: the regularization parameter *c*, a parameter *a* of the linex loss, the kernel parameter σ.
Linex-TSVM: the regularization parameter $c_{1}$, $c_{2}$, $c_{3}$, $c_{4}$, a parameter *a* of the linex loss, the parameters $\varepsilon_{1}$, $\varepsilon_{2}$, $\varepsilon_{3}$, $\varepsilon_{4}$ and the kernel parameter σ.
  where $\varepsilon_{1}=\varepsilon_{2}=\varepsilon_{3}=\varepsilon_{4}=10^{-5}$; $c_{1}$, $c_{2}$, $c_{3}$, $c_{4}:\left\{10^{i}|-5,-4,...,4,5\right\}$; $\sigma ,\varepsilon :\{10^{i}|i=-4,-3,...,3,4\}$. The experimental parameters are selected by ten cross-validation methods, and the test accuracy is the average of 10 clusters of results in each dataset.

#### 4.1.3. Description of the Datasets

**Figure 2:**
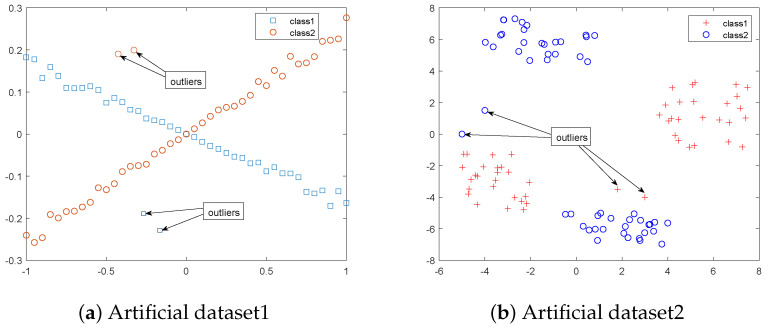
Distribution of artificial datasets with outliers.

To verify the effectiveness of Linex-TSVM, we conduct numerical simulations on different datasets, including seven benchmark datasets from the UCI machine learning repository and two artificial datasets. The datasets are described as follows:

**Artificia datasets:** In the artificial dataset (a) and (b), there are 50 positive samples and 50 negative samples, represented by ‘+’, ‘☐’ and ‘◯’, respectively, as shown in Figure 2. Because the outliers will have a certain impact on the classification performance, it is also the standard to measure the stability of the algorithm. Therefore, we introduce four outliers in the artificial dataset to evaluate its robustness, two of which belong to class +1 and two belong to class −1.

**UCI datasets:** Australian, Spect, Pima, German, Vote, CMC, Sonar, Spect and Large dataset(codrna). Details of the eight UCI datasets are given in Table 1. These UCI datasets are used to test the performance of our algorithms and related algorithms.

We divide all the data sets into ten subsets, including nine training sets and one test set, that is, 10-fold cross-validation, so that the process is repeated ten times, and the average value of the final result is taken as the criterion to measure the performance of the model. At the same time, we normalize the eight participating data sets, which can avoid errors caused by different orders of magnitude and units, keeping the result within [0,1].

### 4.2. Experimental Results on the Employed Datasets without Outliers

Eight UCI datasets are selected and the running results are compared with the other six algorithms to verify the better classification performance of the proposed algorithm. All experimental results presented in Table 2 are based on optimal parameters. Here, ”Time(s)” denotes the average runtime in seconds taken by each algorithm according to the optimal parameters, ”ACC ± S” denotes the average classification accuracy plus or minus standard deviations.

Intuitively, it can be observed from Table 2 that the classification performance of the twin support vector machine based on capped linear loss function proposed in this paper is better than that of the other six models. Except for CMC data sets, Linex-TSVM has better results on other data sets. At the same time, we also observe that the computing time of this model is not dominant, which is because the model is more complex. The time of LSSVM algorithm for solving a system of linear equations is shorter, and compared with SVM, it shortens the time while retaining accuracy, which is in line with the relevant theory. It is worth mentioning that the result of Linex-SVM is still good, which shows that the introduction of linear loss function is meaningful.

Through the detailed analysis of the above experimental results, we can obtain an objective and reasonable conclusion: the use of capped linear loss function on the basis of TBSVM can improve the classification performance, and the introduction of $L_{1}$-norm distance metric can also enhance the robustness of the model; thus, our model is an effective supervision algorithm without the influence of outliers.

### 4.3. Experimental Results on the Employed Datasets with Outliers

#### 4.3.1. Experimental Results on Artificial Dataset with Outliers

**Figure 3:**
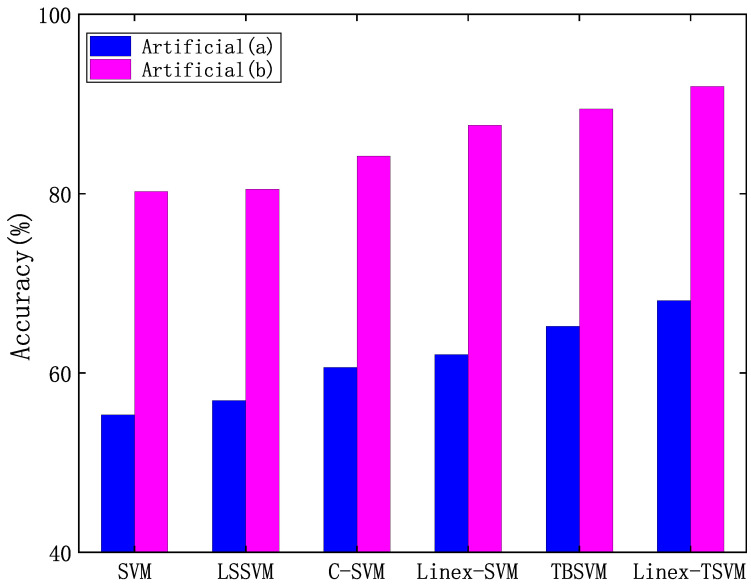
Accuracy of the two artificial datasets with outliers.

It is well known that outliers tend to have a certain impact on classification performance, which is also a measure of the stability of the algorithm. Therefore, we introduce outliers in artificial datasets (a) and (b), respectively, and Figure 2 is displayed visually. In order to further verify the robustness of the capped linear loss function, we show the classification accuracy of this algorithm on artificial data sets (a) and (b) in Figure 3, and compare the other five algorithms.

From Figure 3, we observe that the proposed Linex-TSVM has higher accuracy when considering outliers; on artificial datasets (a) and (b), the classification accuracy of Linex-TSVM is 68.06% and 91.97%, respectively, which is better than the other five algorithms, can deal with outliers well, and has stronger robustness and better classification ability.

In summary, the capped $L_{1}$-norm is robust to different types of outliers in the literature [21,22,23,24,25]. It can overcome the residual error of outliers in the experiment, and can help the model to eliminate the influence of outliers. In particular, the truncated linear loss function in this model can increase the punishment for outliers. In a word, Linex-TSVM can effectively improve the robustness of TBSVM.

#### 4.3.2. Experimental Results on UCI Dataset with Outliers

In order to verify that this model is also suitable for large-scale data with outliers, we add 10% and 25% noises to the eight UCI data sets, respectively. The reason why the algorithm is introduced into the model is that in practical application, there are various kinds of data and there must be different degrees of noise. In order to verify that the model is suitable for data sets of different fields and different sizes to a certain extent, it is necessary to introduce different noise to compare the models. At the same time, we find that after adding noise, the accuracy will fluctuate to a certain extent, but the overall trend shows a slow decline, which shows that when the noise is relatively large, it will have a certain impact on the model, but the model in this paper is more stable. The results, such as Table 3 and Table 4, show that after the introduction of outliers, the seven algorithms all have varying degrees of accuracy fluctuations, but show a downward trend as a whole, and the classification accuracy of Linex-TSVM is almost better than other algorithms. This shows that the model proposed in this paper has stronger robustness.

Specifically, in Table 3 and Table 4, Linex-TSVM has the best accuracy in seven of the eight data sets, while the least squares support vector machine model has the shortest computing time under different noises. It is worth noting that compared with SVM, LSSVM, NPSVM, C-SVM, Linex-SVM, due to the use of capped linear loss function, the penalty for outliers is increased, so it has better classification accuracy. Linex-TSVM is better than Linex-SVM and TBSVM.

**Figure 4:**
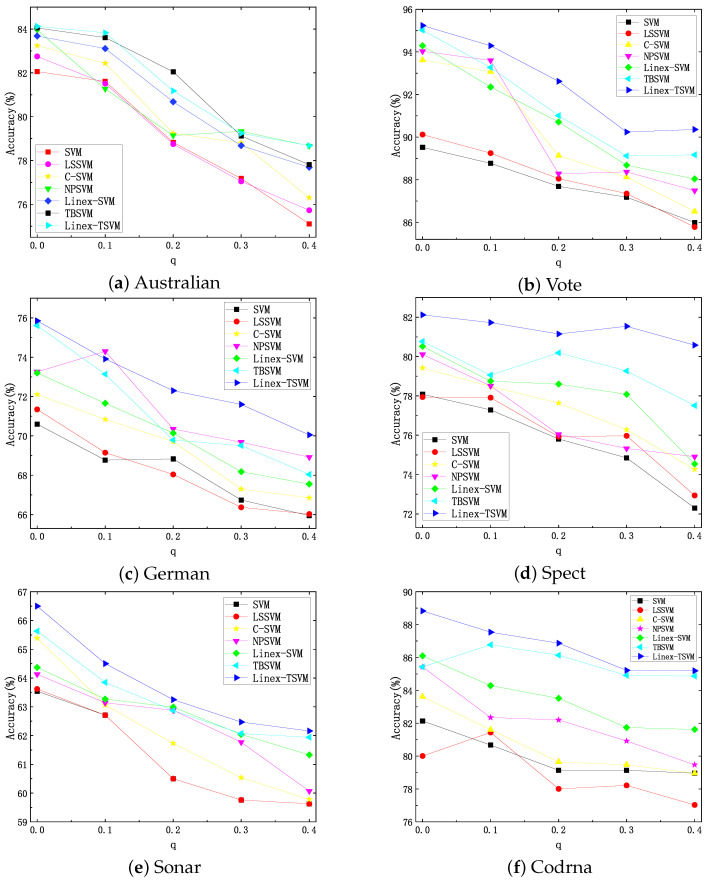
Accuracies of seven algorithms via different noises factors.

Furthermore, in order to more comprehensively analyze the robustness of the algorithm under different noises, we have carried out more experiments on Australian, Spect, Pima, German, Vote, CMC, Sonar, Codrna and Spect, and we use different noises to test the performance of the six algorithms. For an original dataset X, we changed it with $X+\lambda \bar{X}$, where $\lambda =\frac{{q∥X∥}_{F}}{∥\bar{X}{∥}_{F}}$ and *q* is a noise factor. Here, $\bar{X}$ is the noise matrix whose elements are i.i.d. standard Gaussian variables. The value is q∈{0.1,0.2,0.3,0.4}. Through Figure 4, we can observe that under different noise factors, Linex-TSVM shows better classification accuracy and stability, while the other six models are relatively more volatile.

**Figure 5:**
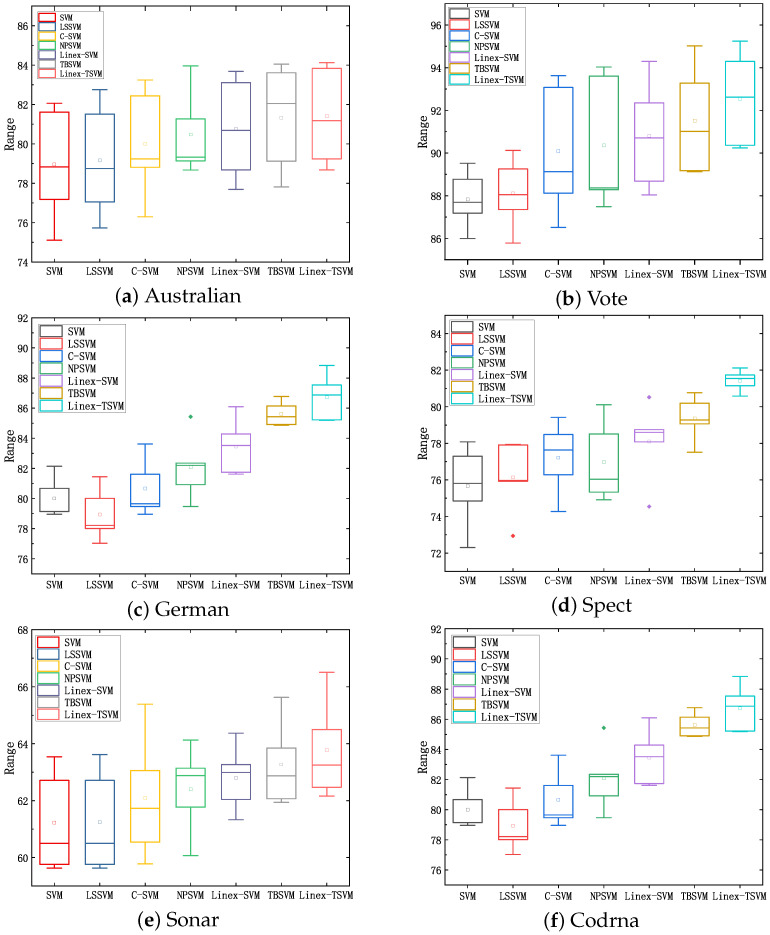
Box diagram of UCI datasets with outliers.

Next, we introduce the box line diagram to verify that the model is better from another point of view. In Figure 5, we select six datasets to analyze the height of the box reflects the fluctuation of the data to a certain extent, that is, it represents the fluctuation of classification accuracy. The upper and lower edges represent the maximum and minimum values of the group of data, and the points outside the box can be understood as "outliers" in the data, so we can directly observe that the classification accuracy of Linex-TSVM is higher than that of other models.

To sum up, the capped linear loss function twin support vector machine proposed in this paper is superior to the other six algorithms in terms of classification accuracy and robustness, indicating that Linex-TSVM is a robust learning algorithm for large-scale data classification with noise.

### 4.4. Analysis for the Convergence

**Figure 6:**
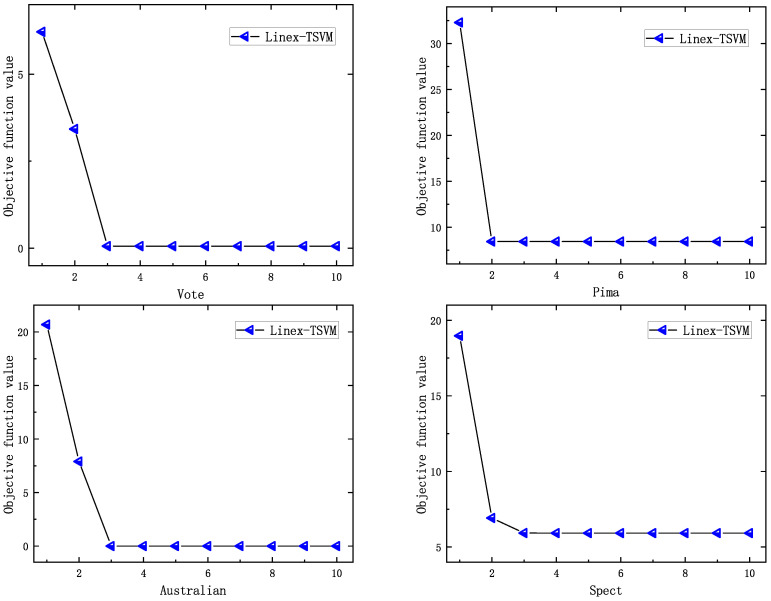
Convergence rate of Linex-TSVM.

In this section, we show the convergence curve of the proposed algorithm on four datasets to directly verify that the convergence speed of the proposed algorithm can achieve the desired speed. The result is Figure 6, where the horizontal axis represents the number of iterations and the vertical axis represents the value of the objective function. We set: when the difference between the target values of two consecutive iterations is less than 0.001 and the number of iterations is less than 50, the iterative process stops.

The result of Figure 6 shows that the value of the objective function of Linex-TSVM decreases monotonously with the increase in the number of iterations, and the algorithm can converge quickly in about 5 iterations, that is, it converges within a limited number of iterations, and we obtain satisfactory results, which is consistent with the previous theoretical analysis.

### 4.5. Statistical Analysis

In this section, the statistical analysis method-Friedman test is used to compare the differences among the six algorithms involved. In this paper, the Friedman test is a statistical test of the homogeneity of multiple (related) samples, which makes full use of all the information in the original data and has many advantages. It is worth noting that the zero hypothesis means that all algorithms have the same performance. When the zero hypothesis is rejected, we can perform the post-processing test of the Nemeny test [26]. Next, the average ranking and accuracy of the six algorithms on seven data sets are shown in Table 5.

Next, we take eight UCI datasets with 10% Gaussian noise as examples to compare the six algorithms. The formula for Friedman statistical variables is as follows:

(32) $$\chi_{F}^{2}=\frac{12N}{k(k+1)}\left[\sum_{i}R_{i}^{2}-\frac{k{\left(k+1\right)}^{2}}{4}\right]=30.18.$$

where *k* is the number of algorithms and *N* is the number of UCI datasets. In our paper, k=7,N=8. $R_{i}$ represents the average ranking of the *i* algorithm on the seven UCI datasets. In addition, according to the $\chi_{F}^{2}$ distribution with k−1 degrees of freedom, we can obtain:

(33) $$F_{F}=\frac{\left(N-1\right)\chi_{F}^{2}}{N\left(k-1\right)-\chi_{F}^{2}}=11.86.$$

where $F_{F}\left(\left(k-1\right),\left(k-1\right)\left(N-1\right)\right)$ obeys the F-distribution, and its degree of freedom is (k−1) and (k−1)(N−1). In this paper, we choose α=0.05 and we can get $F_{\alpha }\left(6,42\right)=2.34$. Obviously, $F_{F}>F_{\alpha }$, we reject the zero hypothesis.

Intuitively, from the Table 5, ee observe that Linex-TSVM has better classification performance, which means that our algorithm is more effective.

Next, through the Nemenyipost-hoctest, we can further compare the errors of the six algorithms in this paper. If the average rank difference between each other is greater than the critical value, the results demonstrate that the performance of the two algorithms is different. By dividing the Studentized range statistic by $\sqrt{2}$, we can obtain $q_{\alpha }=2.95$. Therefore, we calculate the critical difference (CD) by the following formulation:

(34) $$CD=q_{\alpha =0.05}\times \sqrt{\frac{k(k+1)}{6N}}=3.18.$$

**Figure 7:**
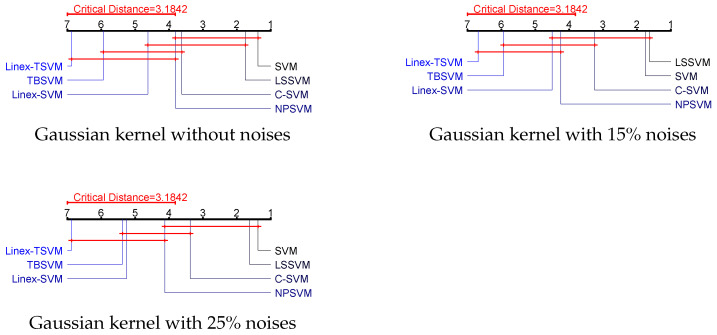
Visualization of post hoc tests for UCI datesets.

Based on Figure 7, the performance of Linex-TSVM is significantly better than SVM, LSSVM, C-SVM, Linex-SVM and TBSVM, but the different between Linex-SVM and TBSVM is not obvious, because the different is smaller than the calculated CD value. Through the above analysis, the Linex-TSVM proposed in this paper has better performance.

## 5. Conclusions

The Twin Support vector machine classification has become a research hotspot. Twin support vector machine models based on different loss functions have been proposed, such as TPMSVM, TWSVM, SG-TSVM and so on. It is urgent to propose a loss function with better performance under the framework of support vector machine. The summary of this paper is as follows:

Firstly, this paper proposes capped linear loss function and applies it to twin support vector machine, and proposes a new robust classification model, which is called truncated linear loss function twin support vector machine. Compared with the linear loss support vector machine model proposed by Ma et al. [8], it has better classification performance. Secondly, we give an efficient iterative algorithm to solve Linex-TSVM. Unlike SVM, which needs to solve a large QP problem, this algorithm needs to solve a pair of small QP problems. Finally, we strictly analyze the computational complexity of the algorithm; it is verified that Linex-SVM satisfies the Bayesian rule. Experimental results on multiple data sets demonstrate that our algorithm Linex-TSVM is more feasible and robust in dealing with large-scale datasets with outliers than other models, and intuitively show the convergence of the algorithm. In particular, compared with SVM, LSSVM, C-SVM, NPSVM, Linex-SVM and TBSVM, the average accuracy of Linex-TSVM is higher than that in the absence of noise. The average accuracy of the model in this paper is higher than that of 4.36%, 4.29%, 2.53%, 2.33%, 1.91% and 0.77%, respectively. Linex-TSVM is more robust and stable for outliers.

The focus of future work is that we should still focus on finding better models to improve different data classification results, shorten the computing time while ensuring accuracy, and extend the model of this paper to other work, such as multi-classification problems. In future work, we can further consider applying different models to practical hot issues, such as face recognition, fingerprint recognition, UAV scheduling and so on. Of course, how to develop a better new algorithm for our Linex-TSVM is also very important.

## Figures and Tables

**Table 1:** Characteristics of UCI datasets.

Datasets	Samples	Attributes	Datasets	Samples	Attributes
Australian	690	14	Spect	267	45
Pima	768	8	German	1000	24
Sonar	198	60	Vote	432	16
CMC	1140	9	codrna	59,535	9

**Table 2:** Experimental results on UCI datasets without noise.

	SVM	LSSVM	C-SVM	NPSVM	Linex-SVM	TBSVM	Linex-TSVM
Datasets	ACC ± S (%)	ACC ± S (%)	ACC ± S (%)	ACC ± S (%)	ACC ± S (%)	ACC ± S (%)	ACC ± S (%)
	Times (s)	Times (s)	Times (s)	Times (s)	Times (s)	Times (s)	Times (s)
Australian	82.06 ± 0.63	82.75 ± 1.52	83.24 ± 3.39	83.96 ± 2.53	83.68 ± 1.63	84.05 ± 1.14	**84.12 ± 0.42**
	1.905	**1.108**	1.241	2.037	2.644	2.358	4.535
Vote	89.52 ± 0.73	90.12 ± 1.17	93.62 ± 1.53	94.03 ± 1.87	94.29 ± 0.97	95.02 ± 1.34	**95.24 ± 1.05**
	0.943	**0.793**	1.832	1.834	1.846	1.115	2.062
German	70.60 ± 1.74	71.35 ± 0.21	72.11 ± 3.22	73.26 ± 1.26	73.20 ± 2.86	75.61 ± 0.87	**75.85 ± 0.21**
	2.286	**1.719**	2.303	3.421	4.672	3.719	9.695
Spect	78.08 ± 1.62	77.94 ± 1.53	79.42 ± 5.25	80.11 ± 0.41	80.52 ± 2.34	80.77 ± 1.09	**82.12 ± 1.01**
	**0.639**	0.945	1.347	2.014	1.739	1.235	1.438
CMC	55.13 ± 3.62	56.42 ± 2.79	57.52 ± 1.52	56.33 ± 3.15	57.17 ± 0.76	**61.12 ± 5.71**	60.97 ± 0.62
	1.441	**1.367**	2.980	3.286	5.862	3.744	8.328
Pima	73.42 ± 0.72	72.79 ± 1.24	74.21 ± 1.03	73.48 ± 1.64	74.63 ± 1.69	75.58 ± 0.66	**75.79 ± 1.31**
	1.117	**1.036**	1.125	2.185	2.092	1.626	5.032
Sonar	63.54 ± 2.54	63.62 ± 2.34	65.39 ± 0.69	64.13 ± 0.68	64.37 ± 1.42	65.63 ± 2.16	**66.50 ± 0.68**
	0.350	**0.137**	0.659	0.517	0.813	0.534	0.913
codrna	82.14 ± 1.73	80.01 ± 2.61	83.62 ± 0.11	85.43 ± 2.24	86.10 ± 0.62	85.43 ± 3.11	**88.73 ± 3.40**
	50.947	**43.400**	49.203	51.229	70.562	66.548	59.914

**Table 3:** Experimental results on UCI datasets with 10% noise.

	SVM	LSSVM	C-SVM	NPSVM	Linex-SVM	TBSVM	Linex-TSVM
Datasets	ACC ± S (%)	ACC ± S (%)	ACC ± S (%)	ACC ± S (%)	ACC ± S (%)	ACC ± S (%)	ACC ± S (%)
	Times (s)	Times (s)	Times (s)	Times (s)	Times (s)	Times (s)	Times (s)
Australian	81.61 ± 0.82	81.51 ± 0.83	82.44 ± 2.60	81.27 ± 1.39	83.11 ± 1.45	83.61 ± 1.07	**83.83 ± 0.31**
	1.866	**1.154**	1.346	1.302	2.842	2.775	4.279
Vote	88.77 ± 0.63	89.25 ± 1.38	93.07 ± 1.63	93.60 ± 2.95	92.35 ± 1.68	93.27 ± 2.17	**94.29 ± 1.01**
	1.193	**0.810**	1.893	1.027	1.088	1.607	2.034
German	68.22 ± 0.89	69.15 ± 2.41	70.84 ± 2.41	**74.30 ± 2.26**	71.66 ± 2.50	73.14 ± 1.21	73.91 ± 0.82
	2.876	**2.274**	2.507	2.460	4.339	3.105	9.148
Spect	77.29 ± 1.87	77.91 ± 0.88	78.48 ± 3.57	78.51 ± 3.11	78.75 ± 1.35	79.06 ± 4.62	**81.73 ± 0.94**
	1.519	**0.988**	1.830	1.616	1.613	1.012	1.910
CMC	54.74 ± 3.30	53.20 ± 2.79	54.59 ± 2.24	58.03 ± 0.87	57.74 ± 1.17	**59.91 ± 0.13**	**59.91 ± 0.75**
	1.441	**1.367**	2.980	4.339	5.862	3.744	8.215
Pima	70.42 ± 0.72	71.79 ± 1.24	72.21 ± 1.03	71.82 ± 0.14	72.70 ± 1.69	**73.92 ± 0.66**	73.53 ± 1.31
	1.535	**1.239**	1.599	3.700	2.272	1.803	5.118
Sonar	62.93 ± 2.54	62.71 ± 2.34	63.06 ± 1.19	63.14 ± 3.01	63.27 ± 1.42	63.85 ± 1.13	**64.50 ± 4.95**
	1.184	0.917	**0.726**	1.715	1.476	1.244	1.619
Codrna	80.67 ± 2.67	81.44 ± 3.61	81.61 ± 1.02	82.35 ± 1.11	84.29 ± 0.90	86.77 ± 1.30	**87.54 ± 0.34**
	54.302	**46.169**	50.495	62.376	55.482	61.517	58.455

**Table 4:** Experimental results on UCI datasets with 25% Gaussian noise.

	SVM	LSSVM	C-SVM	NPSVM	Linex-SVM	TBSVM	Linex-TSVM
Datasets	ACC ± S (%)	ACC ± S (%)	ACC ± S (%)	ACC ± S (%)	ACC ± S (%)	ACC ± S (%)	ACC ± S (%)
	Times (s)	Times (s)	Times (s)	Times (s)	Times (s)	Times (s)	Times (s)
Australian	78.83 ± 1.27	78.75 ± 1.52	79.24 ± 3.39	79.14 ± 1.67	80.68 ± 1.63	**82.05 ± 1.14**	81.18 ± 3.74
	1.365	**1.233**	1.490	1.910	2.076	2.851	4.648
Vote	87.69 ± 1.07	88.05 ± 0.98	89.13 ± 1.39	88.28 ± 2.38	90.71 ± 0.88	91.01 ± 2.59	**92.62 ± 3.37**
	1.053	**0.928**	1.634	1.667	2.069	1.942	2.143
German	68.83 ± 1.21	68.04 ± 0.80	69.71 ± 2.36	70.35 ± 1.64	70.14 ± 2.84	69.79 ± 3.54	**72.30 ± 0.99**
	2.922	**2.421**	2.665	5.196	4.904	3.454	8.615
Spect	75.81 ± 1.17	75.93 ± 0.81	77.64 ± 1.53	76.04 ± 1.30	78.60 ± 2.09	80.19 ± 3.77	**81.15 ± 3.06**
	0.703	**0.914**	0.998	1.632	1.143	1.447	1.519
CMC	52.13 ± 3.62	53.42 ± 2.79	54.52 ± 1.52	54.86 ± 0.88	55.17 ± 0.76	56.91 ± 0.13	**57.79 ± 3.50**
	2.951	**2.566**	3.291	5.157	4.017	4.521	8.693
Pima	70.42 ± 0.72	71.79 ± 1.24	72.16 ± 1.03	73.25 ± 3.67	73.70 ± 1.69	72.43 ± 2.41	**73.92 ± 0.93**
	1.785	**1.355**	1.936	1.902	2.084	2.741	5.375
Sonar	60.24 ± 4.95	60.50 ± 0.36	61.73 ± 1.06	62.88 ± 2.78	62.99 ± 1.12	62.87 ± 0.94	**63.25 ± 0.35**
	0.861	**0.352**	0.886	1.749	1.347	1.365	1.698
codrna	79.14 ± 1.91	78.01 ± 2.76	79.65 ± 3.08	82.20 ± 5.29	83.52 ± 1.63	86.14 ± 2.30	**86.87 ± 1.49**
	58.147	54.990	**53.892**	68.956	70.108	69.560	71.928

**Table 5:** Average accuracy and ranks of six algorithms on UCI datasets with 0%, 10%, 25% Gaussian noise.

	SVM	LSSVM	C-SVM	NPSVM	Linex-SVM	TBSVM	Linex-TSVM
Avg.ACC 0%	74.31	74.38	76.14	76.34	76.76	77.90	78.67
Avg.rank 0%	6.63	6.25	4.38	4.25	3.38	2.00	1.13
Avg.ACC 10%	73.08	73.37	74.54	75.38	75.48	76.69	77.41
Avg.rank 10%	6.38	6.25	4.13	3.88	3.50	1.94	1.31
Avg.ACC 25%	71.64	71.81	72.97	73.38	74.44	75.17	76.14
Avg.rank 25%	6.63	6.38	4.63	4.00	2.88	2.38	1.13
